# Building-Related Environmental Intolerance and Associated Health in the General Population

**DOI:** 10.3390/ijerph15092047

**Published:** 2018-09-19

**Authors:** Kirsi Karvala, Markku Sainio, Eva Palmquist, Anna-Sara Claeson, Maj-Helen Nyback, Steven Nordin

**Affiliations:** 1 Finnish Institute of Occupational Health, 00032 Helsinki, Finland; markku.sainio@ttl.fi; 2 Department of Psychology, Stockholm University, 106 91 Stockholm, Sweden; eva.palmquist@psychology.su.se; 3 Department of Psychology, Umeå University, 901 87 Umeå, Sweden; anna-sara.claeson@umu.se (A.-S.C.); steven.nordin@umu.se (S.N.); 4 Novia University of Applied Sciences, 65200 Vaasa, Finland; maj-helen.nyback@novia.fi

**Keywords:** environmental intolerance, building-related intolerance, sick-building syndrome, asthma, functional somatic syndrome

## Abstract

People frequently attribute adverse symptoms to particular buildings when exposure to pollutants is low, within nonhazardous levels. Our aim was to characterize building-related intolerance (BRI) in the general population. Data were derived from two population-based questionnaire surveys, the Västerbotten and Österbotten Environmental Health Study. We identified cases of BRI if respondents reported symptoms emerging from residing in certain buildings, when most other people had none. The questionnaires covered lifestyle factors, perceived general health, BRI duration and symptom frequency, the emotional and behavioral impact of BRI, coping strategies, and physician-diagnosed diseases. From the total of 4941 participants, we formed two case groups, 275 (5.6%) fulfilled criteria for self-reported BRI, and 123 (2.5%) for BRI with wide-ranging symptoms. Individuals in both case groups were significantly more often female, single, and perceived their general health as poorer than the referents, i.e., those reporting no BRI symptoms. The mean duration of BRI was 12 years. In both case groups, avoidance behavior was found in over 60%, and nearly half of the sample had sought medical care. BRI with wide-ranging symptoms was associated with elevated odds for all studied comorbidities (somatic and psychiatric diseases and functional somatic syndromes). The perceived health of individuals with BRI is poorer and comorbidities are more frequent than among referents. BRI seems to be similar to other environmental intolerances and shares features with functional somatic syndromes.

## 1. Introduction

In modern societies, some people may perceive adverse air quality in certain buildings. They report a broad spectrum of symptoms that occur while residing in the building, and which diminish when away from it [1].

In practice, buildings are usually investigated in an attempt to find causes for the symptoms, and occasionally suspected exposures are found, such as inadequate ventilation, emissions from furnishing and building materials, or moisture and mold in building structures. However, symptoms are not specific to any of the pollutants; they may also occur in buildings in which only minor or no deficiencies are found in indoor-air quality, and are sometimes even resistant to extensive building renovation [2]. Both the type and severity of the symptoms may vary greatly among occupants within the same building. Medical examinations reveal no organ pathology to explain the wide variety of symptoms. Symptoms may persist for years for some individuals, and may lead to significant disability [3,4].

This condition has been difficult to define, as it has been poorly understood from a medical perspective. In 1983, the World Health Organization (WHO) launched the term “sick-building syndrome” (SBS) to describe such nonspecific symptom complexes for which no obvious cause is normally evident [1]. The term is still commonly used in the scientific literature [2], though it has been criticized, one reason being that it is the occupants, rather than the building, that are sick and suffer from symptoms. The latest review defines SBS as “medical symptoms with an unclear cause, but with a possible relation to the indoor environment” [2].

SBS has been widely studied, and symptoms have been shown to associate with environmental, psychosocial, and personal factors [2,5,6]. So far, research focusing on building-related pollutants has been unable to convincingly explain persistent symptoms. As similar observations have been made in regards to other environment-attributed intolerances, for example, multiple chemical sensitivity and electric hypersensitivity, in 1996, the WHO organized a workshop that introduced the concept of idiopathic environmental intolerance (IEI) [7]. The term covers all conditions with heterogeneous somatic symptoms that purportedly arise in response to low-dose factors in an everyday environment, but for which there is no solid evidence of an underlying organic dysfunction with a causal link to the exposure. SBS shares features with IEI and has also been regarded as a form of IEI [8,9]. A common feature of all variants of IEI is that certain environments provoke chronic reactivity to perceived triggers, which then encourages or forces the individual to avoid these environments.

Increasing knowledge of the mechanisms in the development of IEI suggests that the core of intolerance to certain buildings is the chronic responsivity of the individual, rather than the exposure [8,9]. Building-related intolerance (BRI) seems to develop after somatic symptoms first emerge in conditions with more or less poor indoor air quality. A conceivable explanation is that, once symptoms become linked to these particular environmental sources, the perception of specific environmental stimuli fosters the formation of symptoms and maintains them over time, leading to a chronic, complex condition of IEI [8].

In our earlier studies of BRI in the general population, we reported a prevalence rate for self-reported BRI of 4.8% in a Swedish and 7.2% in a Finnish population [10], large comorbidity with various other forms of environmental intolerances [11], airway inflammatory diseases and symptoms [12], and multimorbidity with asthma/allergy and chemical intolerance [13].

Documentation of the characteristic features of BRI in the general population is scarce. However, in certain specific samples, some features have been identified as being associated with BRI, such as the female sex and personality traits among office workers [6,14,15,16,17].

The objective of this study was to obtain a better understanding of BRI in the general population in terms of (i) the role of demographics, lifestyle factors, and perceived general health; (ii) duration, symptom frequency, and emotional and behavioral impact; (iii) coping strategies; and (iv) comorbidity with somatic conditions or diseases, psychiatric conditions, and functional somatic syndromes.

## 2. Materials and Methods

### 2.1. Populations and Samples

We derived our data from the Västerbotten and Österbotten Environmental Health Studies, which are questionnaire-based surveys that investigate various forms of environmental intolerance in Sweden and Finland. Random samples of 8520 and 4606 individuals in the counties of Västerbotten in Sweden (March–April 2010) and Österbotten in Finland (March–April 2012), respectively, were invited to participate. Separated by the Kvarken ocean, the coast-to-coast distance between Västerbotten and Österbotten is about 80 km, and about 25 km between their outmost islands. The samples were stratified for age and sex, with the age strata 18–29, 30–39, 40–49, 50–59, 60–69, and 70–79 years. The questionnaire was sent to the participants with instructions to return it via prepaid postage. Those who did not respond to the first invitation received up to two reminders. Among those invited, 3406 (40.0%) of the Västerbotten sample and 1535 (33.3%) of the Österbotten sample volunteered to participate. Thus, 4941 persons participated in total. Table 1 presents the samples’ age and sex distributions. The questions used for this study were identical in both surveys. The questions on coping strategies were included in the Västerbotten study only.

The Swedish and Finnish studies were conducted in accordance with the Helsinki Declaration, and approved by the Umeå Regional Ethics Board (Dnr 09-171M) and the Ethical Board at Pirkanmaa Hospital District (R12052), respectively. All participants gave their informed consent to participate.

### 2.2. Demographics and Health Issues

All participants responded to questions on demographic and lifestyle factors, perceived general health, and diagnoses given by a physician. These diagnoses included rheumatic disease, back/joint/muscle disorder, atopic dermatitis, asthma, fibromyalgia, irritable-bowel syndrome, tinnitus, migraine, anxiety disorder (generalized anxiety disorder and/or panic disorder), depression, and exhaustion syndrome.

### 2.3. Identification of Building-Related Intolerance

We identified two BRI case groups. One group, referred to as “self-reported BRI,” consisted of participants who responded affirmatively to the question, “Are you getting symptoms from residing in certain buildings (nonspecific building-related symptoms) that you were not getting symptoms from before or that you believe most other people are not getting symptoms from?” The other BRI group, referred to as “self-reported BRI and WHO symptoms,” consisted of participants who responded affirmatively to this question and reported symptoms that, according to the WHO, are common in BRI [1]. In 1983, WHO listed a broad spectrum of nonspecific building-related symptoms consisting of mucosal, skin, and general symptoms [1]. Thus, all participants in the group with self-reported BRI and WHO symptoms were also included in the group with self-reported BRI, and the criteria for the former group can be considered stricter than that for the latter. To fulfill the WHO symptom criteria, the participant had to report at least one mucosal/airway symptom (among the symptoms were eye, nose, or throat irritation, sensation of dry mucous membranes, coughing, hoarseness, wheezing, or chest tightness), at least one skin symptom (erythema or itching), and at least one general symptom (mental fatigue, headache, nausea, dizziness, malaise, lethargy, or concentration difficulties). The symptoms had to have been experienced at least once a week, and to have lasted for at least the past three months. They were assessed using the Environmental Hypersensitivity Symptom Inventory [18]. The participants constituted a referent group if they did not fulfill either of the criteria: (1) responding affirmatively to the question about BRI or (2) responding affirmatively to the question, “Are you getting symptoms from odorous/pungent chemicals (not limited to certain buildings), such as perfumes and cleaning agents, in doses that you were not getting symptoms from before or that you believe most other people are not getting symptoms from?”

### 2.4. Duration, Symptom Frequency, and Emotional and Behavioral Impact of BRI

The participants also responded to questions on the duration (“How long have you suffered from BRI?”) and frequency of BRI-related symptoms (“How often in general do you have these symptoms?”—response alternatives: Daily/Once or a few times a week/Once or a few times a month), and the degree of emotional and behavioral impact when visiting buildings that evoke symptoms (“To what extent are you negatively emotionally/behaviorally affected by visiting buildings that evoke symptoms?”). Both the emotional and behavioral impact were rated on the Environmental Annoyance Scale, which is a category scale with 7 semantic descriptors: “Not at all (0)”, “a little (1)”, “partly (2)”, “pretty much (3)”, “rather much (4)”, “to a large extent (5)”, and “extremely (6)”. This scale has ratio-scale properties, and good reliability and validity [19].

### 2.5. Coping Strategies

We used the following questions on coping strategies, but only gave them to the Västerbotten sample: “Do you actively try to avoid buildings that evoke symptoms?”, “Can you, in most cases, affect the environment that causes the symptoms (e.g., by opening a window)?”, “Have you sought medical care for your building-related intolerance?”, and “Have you received any form of treatment for your building-related intolerance?” Regarding the emergence of the symptoms, we asked: “Did the intolerance start with intense or long-term chemical exposure?”

### 2.6. Statistical Analyses

The two BRI case groups were compared with the referents in terms of demographics, lifestyle factors, and perceived general health using independent *t*-tests and chi-square analyses. We conducted logistic regression analyses to obtain crude and adjusted-odds ratios (ORs) for studying comorbidity in BRI with the various diagnoses. The possible confounders were selected from among factors that have been reported to associate with environmental intolerances (sex, age, and socioeconomic class) or are known to be general risk factors for poorer health. Sex and marital/cohabitating status were used for adjustment, as these differed between the groups. The α level was set at 0.05. Statistical analyses were conducted using SPSS version 23 (IBM SPSS Statistics for Windows, Armonk, NY, USA).

## 3. Results

Of the total of 4941 participants, 275 (5.6%) fulfilled the criteria for self-reported BRI, 123 (2.5%) for self-reported BRI and WHO symptoms, and 4180 (84.6%) for referents, whereas 486 participants fulfilled criteria for neither of the three groups and were excluded from further analyses. All outcome variables of the participants in the self-reported BRI/self-reported BRI and WHO symptoms/referent groups in the Västerbotten (*n* = 165/73/2923, respectively) and the Österbotten (110/50/1257, respectively) samples were statistically compared, separately for each of the two case groups. Despite finding minor differences, the samples were combined. Only the following features differed. Those with self-reported BRI in the Västerbotten sample, compared to those in the Österbotten sample, were older, reported longer duration of BRI, and reported BRI-related symptoms less frequently. Those with self-reported BRI and WHO symptoms in the Västerbotten sample also reported BRI-related symptoms less frequently.

Table 2 shows the results of demographics, lifestyle factors, and perceived general health, duration, symptom frequency, and emotional and behavioral impact. Both BRI case groups, in comparison to the referent group, consisted of a significantly larger proportion of women, and a smaller proportion of participants who were married or cohabiting and reported significantly poorer perceived general health. Neither of the two case groups differed significantly from the referent group in terms of age, university education, smoking, or physical exercise. The case groups reported having had BRI for about 12 years on average. About 43% to 53% of these reported BRI-related symptoms either daily or weekly. On the Environmental Annoyance Scale [18], the average ratings of the negative emotional and behavioral impact of visiting buildings that evoked symptoms were positioned between the semantic descriptors “partly” and “pretty much”. Independent t-tests showed that those with daily or weekly BRI-related symptoms, compared to those with monthly symptoms, reported that visiting buildings that evoke symptoms had a significantly more negative emotional (mean = 2.76, SD = 1.72 vs. mean = 2.14, SD = 1.55, *t* = 7.37, *p* < 0.001) and behavioral (mean = 2.31, SD = 1.83 vs. mean = 1.95, SD = 1.50, *t* = 4.18, *p* < 0.001) impact.

**Figure 1:**
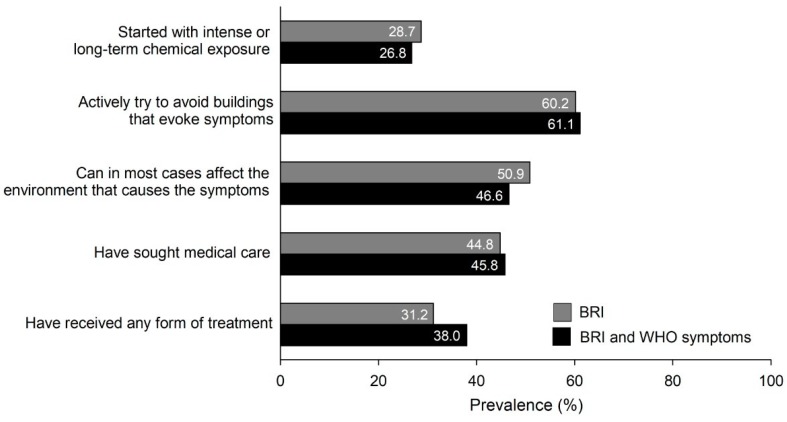
Prevalence (%) of aspects of emergence of BRI and coping strategies in case groups with self-reported BRI (*n* = 275) and with self-reported BRI and BRI-related symptoms according to the WHO (WHO symptoms) (*n* = 123).

Figure 1 shows the results regarding the emergence of symptoms and coping strategies (based on data from the Västerbotten sample only). Slightly more than one-fourth of the BRI cases reported that their intolerance began with intense or long-term chemical exposure. A majority reported actively trying to avoid buildings that evoke symptoms, and about half reported that in most cases they could affect the environment that causes the symptoms. Somewhat less than half of the cases reported having sought medical care, and about one-third that they had received treatment for their BRI.

**Figure 2:**
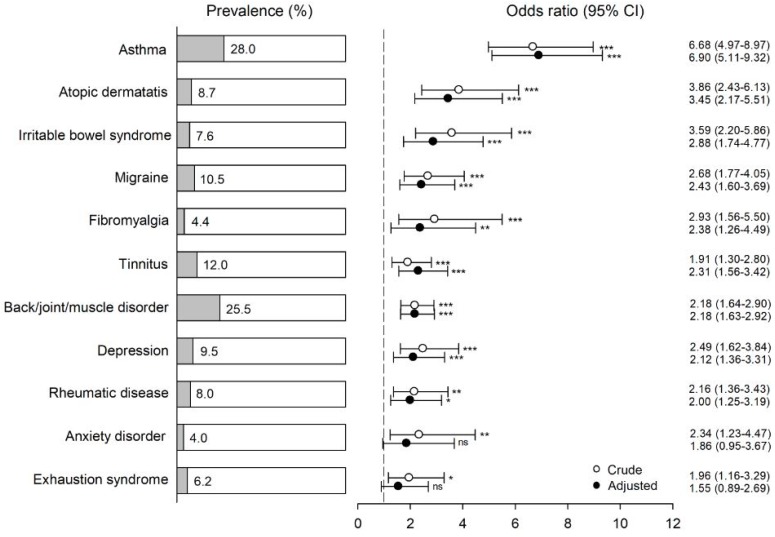
Percentage of physician-based diagnoses among participants with self-reported building-related intolerance (single-item question on getting symptoms, when most other people do not, from residing in a certain building; *n* = 275). Odds ratios (ORs), confidence intervals (CIs), and *p*-values (* *p* < 0.05, ** *p* < 0.01, *** *p* < 0.001, and ^ns^ nonsignificant) for comorbidity with these conditions are given as both unadjusted and adjusted for sex and marriage/cohabiting. Referents were used as a reference group (*n* = 4180). The vertical dashed line represents an OR of unity.

**Figure 3:**
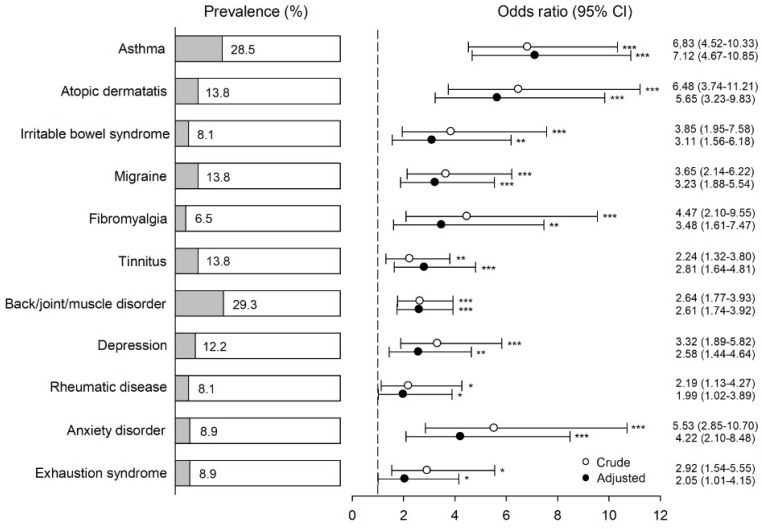
Percentage of physician-based diagnoses among participants with self-reported BRI (based on a single-item question on getting symptoms, when most other people do not, from residing in a certain building) and BRI-related symptoms according to the WHO (*n* = 123). ORs, CIs, and *p*-values (* *p* < 0.05, ** *p* < 0.01, and *** *p* < 0.001) for comorbidity with these conditions are given as both unadjusted and adjusted for sex and marriage/cohabiting. Referents were used as a reference group (*n* = 4180). The vertical dashed line represents an OR of unity.

The percentage of participants who met the criteria for BRI and for BRI and WHO symptoms, and who also had a specific physician-based diagnosis, is shown in Figure 2 and Figure 3, respectively. The figures also show odds ratios (ORs) for comorbidity in the two BRI case groups with various diagnoses as both unadjusted (crude) and adjusted for sex and marital/cohabitation status. With the exception of anxiety disorder and exhaustion syndrome in the BRI group when adjusted, all ORs were significantly greater than unity for all diagnoses, both crude and adjusted. When adjusted, these ORs ranged from 2.00 to 6.90 (1.96 to 6.68 when unadjusted) in the BRI group, and from 1.99 to 7.12 (2.19 to 6.48 when unadjusted) in the BRI and WHO symptoms group.

## 4. Discussion

In this population-based study, we found that individuals with BRI are significantly more often female, single, and perceive their general health as poorer than the referents. We found no overall difference in terms of age, education level, or lifestyle factors (smoking and physical exercise). However, BRI was associated with a low education level specifically among women. We found BRI to be a long-lasting condition, with a mean of 12 years. Of those who had BRI with wide-ranging symptoms (mucosal/airway, skin, and general symptoms), over 50% reported that symptoms occurred daily or weekly. Avoidance behavior was found in over 60%, and nearly half of the sample had sought medical care. BRI with wide-ranging symptoms was associated with elevated odds of all studied comorbidities, both somatic and psychiatric diseases and functional somatic syndromes (adjusted ORs = 2.0–7.1).

Our findings connect BRI to other environmental intolerances. Similar multimorbidity has been reported for environmental intolerance, regardless of the type of exposure in question, as that for chemical intolerance (or multiple chemical sensitivity) [20,21], electromagnetic hypersensitivity [22], and sound intolerance [23]. Most observations concern chemical intolerance, in which psychiatric comorbidity is most often reported [21], but somatic comorbidity and concurrent functional somatic syndromes have also been reported [20]. Thus, nonspecific morbidity may contribute to the development of environmental intolerance.

After adjustments, the highest odds, about sevenfold, were found for asthma in both BRI case groups. An association between chemical intolerance and asthma has been previously found [13,24]. The nature of asthma might provide an explanation for why individuals with asthma seem to be especially vulnerable to the development of environmental intolerance. It is characteristic of asthma that symptoms often occur in response to environmental inhalant triggers, either allergenic or nonallergenic. If the presence of triggers cannot be observed directly, it may be inferred from even harmless contextual cues (such as odor) [25,26]. For an asthmatic, the situation may be interpreted, typically unconsciously, as a health threat initiating biological processes that lead to asthmatic reactions including bronchoconstriction [27]. In the next phase, environmental stimuli may act as a nocebo driven by prior negative expectations and beliefs. The current understanding is that the development of environmental intolerance is driven by similar learning mechanisms: pairings of particular environmental stimuli with symptom experiences leads to automatic stress responses upon perceived exposure [8]. Thus, asthma symptoms can be paired with environmental stimuli and may increase the risk of BRI and other environmental intolerances.

Similarly, symptoms of other comorbidities may become misattributed to environmental factors and lead to the development of BRI. Studies on the co-occurrence of various diseases with environmental intolerances have been studied in cross-sectional study designs, but longitudinal studies are scarce. In a German multicenter study, psychiatric disorders preceded chemical intolerance by several years in a majority of patients [21], which suggests that increased morbidity could be a risk factor of environmentally attributed symptoms.

Wide comorbidity with somatic and mental diseases and broad symptom spectrum are characteristic of all functional somatic syndromes (medically unexplained physical symptoms) [28]. The comorbidity we observed might indicate that environmental intolerances, including BRI, share similar predisposing and perpetuating factors with functional somatic syndromes. Central sensitization provides a plausible explanatory concept as a common mechanism behind the conditions [8,29]. Both environmental intolerance and functional somatic syndromes have shown to activate nonspecific stress responses [30]. Thus, any symptom or dysfunction may increase the risk of environmental factors becoming linked with a negative valence (nocebo) that activates stress mechanisms in the brain and thus predisposes to chronic responsivity such as BRI.

Among our respondents, less than 30% reported that their BRI had begun from intense or long-term chemical exposure. Whether exposures per se explain environmental intolerance has been questioned. Potentially harmful exposures in indoor environments, such as dampness-related agents or volatile organic compounds at levels exceeding sensory-irritation thresholds, may explain transient respiratory or mucosal symptoms, including aggravation of asthma symptoms [31,32]. With the mechanisms discussed above, symptom experiences, with or without exposures, may act as triggers and initiate the development of environmental intolerance. A hallmark of environmental intolerance, i.e., long-lasting reactivity in response to actual as well as sham exposure (nocebo), is more likely to be explained by brain functions than by exposures [8,9].

For an individual with BRI, avoiding perceived exposure is a natural attempt to relieve symptoms. We found avoidance behavior to be common, as over 60% in both BRI case groups reported actively trying to avoid buildings that evoke symptoms. Being forced to avoid certain buildings due to symptoms may increase experiences of disability. It has been proposed that higher degrees of cognitive and behavioral avoidance predict worse long-term outcomes in anxiety disorders and chronic pain [33,34]. Avoidance may strengthen conditioned fear responses, which leads to enhanced responsivity to environmental triggers and illness behavior. However, deconditioning a fear response requires that the individual feels in control of the exposure situation.

In addition to comorbidity, the individuals with BRI in our study perceived their general health as significantly poorer than referents. Earlier, a high number of somatic symptoms, which is a characteristic of environmental intolerance [35], has been associated with poor health status, independently of the etiology of symptoms [36]. Co-occurrence of symptoms (dizziness, pain in legs, respiratory distress, and tiredness) has also proven to be an important predictor of poor health status and limitations due to physical health [37]. Moreover, according to a Dutch study, individuals who attribute somatic symptoms to environmental factors experience poorer health, increased illness behavior, and more severe symptoms than patients with nonspecific physical symptoms and no environmental intolerance [38].

We found that those with frequent (daily or weekly) BRI-related symptoms were more emotionally and behaviorally affected than those with less frequent symptoms. About half of the BRI sufferers experienced that they could not affect the environment that causes their symptoms, which may increase helplessness. Nearly half of the sample had sought medical care, and a third had received some form of treatment. These findings reflect the disability associated with BRI and the need for healthcare services.

The mean duration of BRI was surprisingly long—12 years in both case groups. Environmental intolerance, including BRI [2], is regarded as a persistent state. Our findings are in line with a previous study on chemical intolerance, which showed that, after a nine-year follow-up, most individuals were still symptomatic and retained their illness beliefs [39]. The long duration of the intolerance reflects the chronicity of the condition, and an altered responsiveness in the individual, although fluctuations over time due to, for example, BRI to other intolerances and back to BRI do exist [40].

Our study is one of the few population-based studies on the current subject, which is a strength. Despite its large sample size, a limitation of the study is its moderately low response rate, in particular among young men (see Table 1), which might have resulted in a selection bias. Individuals with symptoms or health problems under investigation were more likely to respond. For example, the high prevalence of asthma (28%) among individuals with BRI might be partly explained by participation bias. On the other hand, the high prevalence of asthma among individuals with environmental intolerance is an expected finding (discussed above). These findings may have exaggerated the role of comorbidity if the healthier individuals among those without environmental intolerance were more willing to participate. However, we regard it as unlikely that selection bias would explain a considerable part of the discovered associations. As our study was cross-sectional, we cannot draw conclusions about causality for the associations observed between the disorders. The lack of an objective or self-reported assessment of indoor environments may be regarded as another limitation. However, we did not include exposure assessment in this study, because the focus was on the association between self-reported intolerance and health-related outcomes, not the effect of exposure on BRI or health. We used a one-item question to define BRI, which may be considered a limitation, but is nonetheless line with most other studies of environmental intolerance [10].

## 5. Conclusions

In conclusion, our findings show that BRI shares features with other environmental intolerances, such as chemical intolerance and electromagnetic hypersensitivity, and thus is a form of environmental intolerance. A similar comorbidity profile with functional somatic syndromes suggests similar mechanisms, and similar predisposing and perpetuating factors for the conditions play a role. When regarding comorbidities, such as asthma, as a consequence of exposure, it should be taken into account that, instead, they might be risk factors for environmental intolerance. BRI is associated with poor perceived health and comorbidities, which may lead to contact with healthcare. The parallels with functional somatic syndromes suggest that conditions might respond to similar interventions, such as active behavioral interventions, which focus on encouraging patients to challenge distorted cognitions and change harmful patterns of behavior [41].

## Figures and Tables

**Table 1:** Numbers of respondents (and response rate) across age and sex strata for the two samples.

Age (Years)	Västerbotten		Österbotten	
	Women	Men	Women	Men
18–29	307 (32.1)	179 (17.3)	128 (28.6)	70 (14.2)
30–39	266 (40.3)	177 (24.7)	121 (36.0)	80 (21.3)
40–49	288 (40.5)	230 (31.0)	140 (37.4)	80 (19.7)
50–59	367 (50.9)	295 (39.5)	192 (46.0)	123 (29.5)
60–69	405 (58.4)	356 (50.7)	186 (44.2)	169 (39.5)
70–79	265 (53.8)	271 (63.9)	131 (44.9)	115 (43.5)
Total sample	1898 (45.2)	1508 (34.9)	898 (39.7)	637 (27.2)

**Table 2:** Description of BRI case groups and referent group in terms of demographics, lifestyle, perceived general health, and comparisons between case groups and referents using t-test and chi-square analysis. Further description of the case groups is also given.

Variable	Self-Reported BRI ^1^	Self-Reported BRI and World Health Organization (WHO) Symptoms ^2^	Referents
	(*n* = 275)	(*n* = 123)	(*n* = 4180)
Age (years), mean (SD)	51.8 (14.5) ^ns^	51.3 (14.4)	51.4 (16.9)
Women, *n* (%)	201 (73.1) ***	96 (78.0) ***	2259 (54.0)
Married/cohabitant, *n* (%)	185 (67.3) *	78 (63.4) *	3098 (74.1)
No response	4 (1.5)	2 (1.6)	34 (0.8)
University education, *n* (%)	121 (44.0) ^ns^	50 (40.7) ^ns^	1614 (38.6)
No response	4 (1.5)	2 (1.6)	71 (1.7)
Smoking, *n* (%)	28 (10.2) ^ns^	15 (12.2) ^ns^	376 (9.0)
No response	0 (0)	0 (0)	29 (0.7)
Physical exercise, *n* (%)			
Once a month or less	37 (13.5) ^ns^	15 (12.2) ^ns^	601 (14.4)
2–4 times/month	53 (19.3)	25 (20.3)	913 (21.8)
2–3 times/week	101 (36.7)	47 (38.2)	1564 (37.4)
More than 3 times/week	79 (28.7)	33 (26.8)	1035 (24.8)
No response	5 (1.8)	3 (2.4)	67 (1.6)
Perceived general health, *n* (%)			
Excellent/very good	75 (27.3) ***	27 (22.0) ***	1665 (39.8)
Good	81 (29.5)	31 (25.2)	1449 (34.7)
Fairly good/poor	118 (42.9)	65 (52.8)	1019 (24.4)
No response	1 (0.4)	0 (0)	47 (1.1)
Duration of BRI (years), mean (SD)	12.4 (11.6)	12.8 (12.3)	
Frequency of BRI-related symptoms			
Daily	55 (20.0)	32 (26.0)	
Weekly	64 (23.3)	33 (26.8)	
Monthly	129 (46.9)	51 (41.5)	
No response	27 (9.8)	7 (5.7)	
Degree of negative impact of visiting buildings that evoke symptoms, mean (SD)			
Emotionally	2.43 (1.68)	2.85 (1.66)	
Behaviorally	2.13 (1.71)	2.51 (1.73)	

^1^ BRI = Building-related intolerance; ^2^ WHO symptoms = Important BRI symptoms according to WHO (1983). * *p* < 0.05, *** *p* < 0.001, ^ns^ nonsignificant.

## References

[1] World Health Organization (WHO) (1983). Indoor Air Pollutants: Exposure and Health Effects. Euro Reports and Studies, 78.

[2] Norbäck D. (2009). An update on sick building syndrome. Curr. Opin. Allergy Clin. Immunol..

[3] Edvardsson B., Stenberg B., Bergdahl J., Eriksson N., Linden G., Widman L. (2008). Medical and social prognoses of non-specific building-related symptoms (Sick Building Syndrome): A follow-up study of patients previously referred to hospital. Int. Arch. Occup. Environ. Health.

[4] Söderholm A., Öhman A., Stenberg B., Nordin S. (2016). Experience of living with nonspecific building-related symptoms. Scand. J. Psychol..

[5] Tietjen G.E., Khubchandani J., Ghosh S., Bhattacharjee S., Kleinfelder J. (2012). Headache symptoms and indoor environmental parameters: Results from the EPA BASE study. Ann. Indian Acad. Neurol..

[6] Lu C.Y., Tsai M.C., Muo C.H., Kuo Y.H., Sung F.C., Wu C.C. (2017). Personal, Psychosocial and Environmental Factors Related to Sick Building Syndrome in Official Employees of Taiwan. Int. J. Environ. Res. Public Health.

[7] IPCS/WHO (1996). Conclusions and recommendations of a workshop on Multiple Chemical Sensitivities (MCS). International Program on Chemical Safety/World Health Organization. Regul. Toxicol. Pharmacol..

[8] Van den Bergh O., Brown R., Petersen S., Witthöft M. (2017). Idiopathic Environmental Intolerance: A Comprehensive Model. Clin. Psychol. Sci..

[9] Hetherington L., Battershill J. (2013). Review of evidence for a toxicological mechanism of idiopathic environmental intolerance. Hum. Exp. Toxicol..

[10] Karvala K., Sainio M., Palmquist E., Nyback M.H., Nordin S. (2018). Prevalence of various environmental intolerances in a Swedish and Finnish general population. Environ. Res..

[11] Palmquist E., Claeson A.S., Neely G., Stenberg B., Nordin S. (2014). Overlap in prevalence between various types of environmental intolerance. Int. J. Hyg. Environ. Health.

[12] Claeson A.S., Andersson H., Wikdahl F., Nyback M.H., Nordin S. (2018). Comorbidity of airway inflammatory diseases in chemical and building-related intolerance. J. Occup. Environ. Med..

[13] Lind N., Söderholm A., Palmquist E., Andersson L., Millqvist E., Nordin S. (2017). Comorbidity and Multimorbidity of Asthma and Allergy and Intolerance to Chemicals and Certain Buildings. J. Occup. Environ. Med..

[14] Björnsson E., Janson C., Norbäck D., Boman G. (1998). Symptoms related to the sick building syndrome in a general population sample: Associations with atopy, bronchial hyper-responsiveness and anxiety. Int. J. Tuberc. Lung Dis..

[15] Eriksson N.M., Stenberg B.G. (2006). Baseline prevalence of symptoms related to indoor environment. Scand. J. Public Health.

[16] Gomzi M., Bobic J., Radosevic-Vidacek B., Macan J., Varnai V.M., Milkovic-Kraus S., Kanceljak-Macan B. (2007). Sick building syndrome: Psychological, somatic, and environmental determinants. Arch. Environ. Occup. Health.

[17] Brasche S., Bullinger M., Morfeld M., Gebhardt H.J., Bischof W. (2001). Why do women suffer from sick building syndrome more often than men?—Subjective higher sensitivity versus objective causes. Indoor Air.

[18] Nordin S., Palmquist E., Claeson A.S., Stenberg B. (2013). The environmental hypersensitivity symptom inventory: Metric properties and normative data from a population-based study. Arch. Public Health.

[19] Nordin S., Liden E., Gidlöf-Gunnarsson A. (2009). Cognition and neurosciences development and evaluation of a category ratio scale with semantic descriptors: The Environmental Annoyance Scale. Scand. J. Psychol..

[20] Bell I.R., Miller C.S., Schwartz G.E., Peterson J.M., Amend D. (1996). Neuropsychiatric and somatic characteristics of young adults with and without self-reported chemical odor intolerance and chemical sensitivity. Arch. Environ. Health.

[21] Eis D., Helm D., Muhlinghaus T., Birkner N., Dietel A., Eikmann T., Gieler U., Herr C., Lacour M., Nowak D. (2008). The German Multicentre Study on Multiple Chemical Sensitivity (MCS). Int. J. Hyg. Environ. Health.

[22] Johansson A., Nordin S., Heiden M., Sandström M. (2010). Symptoms, personality traits, and stress in people with mobile phone-related symptoms and electromagnetic hypersensitivity. J. Psychosom. Res..

[23] Paulin J., Andersson L., Nordin S. (2016). Characteristics of hyperacusis in the general population. Noise Health.

[24] Caress S.M., Steinemann A.C. (2009). Asthma and chemical hypersensitivity: Prevalence, etiology, and age of onset. Toxicol. Ind. Health.

[25] Janssens T., Ritz T. (2013). Perceived triggers of asthma: Key to symptom perception and management. Clin. Exp. Allergy.

[26] Jaen C., Dalton P. (2014). Asthma and odors: The role of risk perception in asthma exacerbation. J. Psychosom. Res..

[27] Chen E., Miller G.E. (2007). Stress and inflammation in exacerbations of asthma. Brain Behav. Immun..

[28] Park J., Gilmour H. (2017). Medically unexplained physical symptoms (MUPS) among adults in Canada: Comorbidity, health care use and employment. Health Rep..

[29] Yunus M.B. (2015). Editorial review: An update on central sensitivity syndromes and the issues of nosology and psychobiology. Curr. Rheumatol. Rev..

[30] Dantoft T.M., Skovbjerg S., Andersson L., Claeson A.S., Lind N., Nordin S., Brix S. (2015). Inflammatory Mediator Profiling of n-butanol Exposed Upper Airways in Individuals with Multiple Chemical Sensitivity. PLoS ONE.

[31] Kanchongkittiphon W., Mendell M.J., Gaffin J.M., Wang G., Phipatanakul W. (2015). Indoor environmental exposures and exacerbation of asthma: An update to the 2000 review by the Institute of Medicine. Environ. Health Perspect..

[32] Wolkoff P. (2013). Indoor air pollutants in office environments: Assessment of comfort, health, and performance. Int. J. Hyg. Environ. Health.

[33] Leeuw M., Goossens M.E., Linton S.J., Crombez G., Boersma K., Vlaeyen J.W. (2007). The fear-avoidance model of musculoskeletal pain: Current state of scientific evidence. J. Behav. Med..

[34] Beesdo-Baum K., Jenjahn E., Hofler M., Lueken U., Becker E.S., Hoyer J. (2012). Avoidance, safety behavior, and reassurance seeking in generalized anxiety disorder. Depress. Anxiety.

[35] Dantoft T.M., Andersson L., Nordin S., Skovbjerg S. (2015). Chemical intolerance. Curr. Rheumatol. Rev..

[36] Tomenson B., Essau C., Jacobi F., Ladwig K.H., Leiknes K.A., Lieb R., Meinlschmidt G., McBeth J., Rosmalen J., Rief W. (2013). Total somatic symptom score as a predictor of health outcome in somatic symptom disorders. Br. J. Psychiatry.

[37] Eliasen M., Kreiner S., Ebstrup J.F., Poulsen C.H., Lau C.J., Skovbjerg S., Fink P.K., Jørgensen T. (2016). Somatic Symptoms: Prevalence, Co-Occurrence and Associations with Self-Perceived Health and Limitations Due to Physical Health—A Danish Population-Based Study. PLoS ONE.

[38] Baliatsas C., van Kamp I., Hooiveld M., Yzermans J., Lebret E. (2014). Comparing non-specific physical symptoms in environmentally sensitive patients: Prevalence, duration, functional status and illness behavior. J. Psychosom. Res..

[39] Black D.W., Okiishi C., Schlosser S. (2001). The Iowa follow-up of chemically sensitive persons. Ann. N. Y. Acad. Sci..

[40] Palmquist E. (2017). Environmental Intolerance: Psychological Risk and Health Factors. Ph.D. Thesis.

[41] van Dessel N., den Boeft M., van der Wouden J.C., Kleinstäuber M., Leone S.S., Terluin B., Numans M.E., van der Horst H.E., van Marwijk H. (2014). Non-pharmacological interventions for somatoform disorders and medically unexplained physical symptoms (MUPS) in adults. Cochrane Database Syst. Rev..

